# A54 THE IMPACT OF NON-INVASIVE TRANSCUTANEOUS AURICULAR VAGUS NERVE STIMULATION (NITAVNS) ON THE DEVELOPMENT OF EXPERIMENTAL COLITIS IN MICE

**DOI:** 10.1093/jcag/gwac036.054

**Published:** 2023-03-07

**Authors:** F Hesampour, D Tshikudi, A Veysel Özden, C N Bernstein, J -E Ghia

**Affiliations:** 1 Immunology, University of Manitoba, Winnipeg, Canada; 2 Bahcesehir University; 3 Vagustim, Istanbul, Türkiye; 4 Internal Medicine; 5 Inflammatory Bowel Disease Clinical & Research Centre, University of Manitoba; 6 Children’s Hospital Research Institute of Manitoba, Winnipeg, Canada

## Abstract

**Background:**

The anti-inflammatory impacts of stimulating the vagus nerve as a part of the gut-brain axis on intestinal macrophage activity and inflammatory cytokine production have been documented. While vagus nerve stimulation until now has been chiefly invasive, non-invasive auricular vagus nerve stimulation (NitaVNS) has no adverse effects associated with invasive stimulation. The NitaVNS-mediated activation of brainstem nuclei can exert anti-inflammatory effects directly on the colon or indirectly through the spleen via a macrophage-acetylcholine-dependent pathway.

**Purpose:**

To examine the effects of NitaVNS on the development of acute colitis in mice using a preclinical model.

**Method:**

Thirty C57BL/6 mice aged 11 to 12 weeks were divided into four groups: control (CTRL)-NitaVNS, CTRL-Sham, dextran sulphate sodium (DSS)-NitaVNS and DSS-Sham. Pre-set stimulation parameters (10 minutes duration, 10 V voltage, 500 s pulse width, and 20 Hz frequency) were applied for NitaVNS or sham-paw stimulation. 5% DSS was administered to induce colitis for five consecutive days, whereas CTRL mice received regular water. Weight loss, stool consistency, blood in the feces, and disease activity index were evaluated daily. Rectal bleeding, colon bleeding, and fecal consistency were determined as a composite macroscopic score upon sacrifice. Collected colon and blood samples were assessed for pro-inflammatory parameters such as interleukin (IL)-1β, tumor necrosis factor (TNF)-α, IL-6, C-reactive protein (CRP), matrix metalloproteinase (MMP)-2, macrophage inflammatory protein (Mip)-1α and β, and anti-inflammatory cytokine tumor growth factor (TGF)-β using qRT-PCR and ELISA.

**Result(s):**

Compared to colitic-sham mice, colitic NitaVNS mice demonstrated a delayed onset and a significantly lower clinical disease severity. Colitis induced significant weight loss, rectal bleeding, and stool consistency loss, and NitaVNS significantly abolished these effects. In colitic conditions, NitaVNS decreased the disease activity index significantly compared to the colitic sham group. Colitis increased the macroscopic scores, and NitaVNS decreased them significantly. Conversely, colon length was significantly reduced in colitic conditions, but NitaVNS abrogated that deleterious effect. mRNA expression levels of IL-1β and TNF-α were significantly increased in colitic mice and NitaVNS decreased them significantly. Whereas Mip-1α and 1β were not modified in colitic conditions, NitaVNS significantly decreased these markers. MMP2 mRNA expression and colonic IL-6 and IL-1β protein levels were not altered in all conditions. However, colitis induced a significant decrease in colonic TGF-β levels, but NitaVNS did not amplify that effect. Interestingly, in the serum of colitic mice, NitaVNS significantly increased and decreased TGF-β and CRP levels, respectively.

**Conclusion(s):**

These findings suggest that NitaVNS possess some spatial anti-inflammatory effects in acute colitis through the up or downregulation of anti- or pro-inflammatory markers.

**Disclosure of Interest:**

F. Hesampour: None Declared, D. Tshikudi: None Declared, A. Veysel Özden Employee of: Company providing the stimulator, C. N. Bernstein: None Declared, J.-E. Ghia: None Declared

